# Development and validation of an intra-tumoral and peri-tumoral radiomics model based on dynamic contrast-enhanced ultrasound for predicting lymph node metastasis in type 2 diabetic patients with thyroid cancer

**DOI:** 10.3389/fendo.2026.1763631

**Published:** 2026-06-23

**Authors:** Siyao Li, Yunzhi Zhou, Wanyan Li, Ansheng Liu, Yanjun Hu, Chaoxue Zhang, Yayang Duan

**Affiliations:** 1 Department of Ultrasound, The Second Affiliated Hospital of Anhui Medical University, Hefei, Anhui, China; 2 Department of Ultrasound, The Affiliated Yantai Yuhuangding Hospital of Qingdao University, Yantai, Shandong, China; 3 Department of Medical Imaging, First Clinical School of Medicine, Anhui Medical University, Hefei, Anhui, China; 4 Department of Ultrasound, Linquan County People’s Hospital, Fuyang, Anhui, China; 5 Department of Ultrasound, The First Affiliated Hospital of Anhui University of Chinese Medicine, Hefei, Anhui, China; 6 Department of Medical Imaging, Cancer Hospital of China Medical University, Liaoning Cancer Hospital & Institute, Shenyang, Liaoning, China; 7 Department of Ultrasound, The First Affiliated Hospital of Anhui Medical University, Hefei, Anhui, China

**Keywords:** contrast-enhanced ultrasound, lymph node metastasis, radiomics, thyroid cancer, type 2 diabetes

## Abstract

**Objectives:**

This study aimed to develop and validate intra-tumoral and peri-tumoral radiomics models based on dynamic contrast-enhanced ultrasound (CEUS) to preoperatively predict lymph node metastasis (LNM) in thyroid cancer patients with type 2 diabetes.

**Materials and methods:**

A total of 203 pathologically confirmed diabetic thyroid cancer patients from three centers were retrospectively included and divided into a training cohort and two external validation cohorts. Radiomics features were extracted from CEUS parameters—time to enhancement (TTE), time to half-peak (TTHP), time to peak (TTP), and washout time (WT). Feature dimensionality reduction was performed using Variance Threshold, SelectKBest, and LASSO regression. Key LNM-related features were screened in the training cohort, and the optimal peri-tumoral region (1 mm vs 2 mm) was determined. Intra-tumoral and peri-tumoral radiomics scores were constructed and integrated into a multivariate logistic regression model. Model performance, calibration, and clinical utility were evaluated across all cohorts.

**Results:**

The 2 mm peri-tumoral region yielded a higher AUC than the 1 mm region in all cohorts, with statistical significance in the training cohort and a consistent trend in the external validation cohorts. The combined radiomics model achieved AUCs of 0.930 (95% CI: 0.876–0.964), 0.907 (95% CI: 0.796–0.968), and 0.865 (95% CI: 0.739–0.941) in the training and external validation cohorts. Calibration curves showed good agreement between predicted and actual outcomes, and decision curve analysis demonstrated substantial clinical benefit.

**Conclusions:**

The CEUS-based combined radiomics model using intra-tumoral and 2 mm peri-tumoral features provides an effective tool for preoperative LNM prediction in thyroid cancer patients with type 2 diabetes.

## 1 Introduction

Thyroid cancer is the most common endocrine malignancy, with its incidence increasing rapidly over the past few decades. It is the fastest-growing cancer worldwide and is projected to become the fourth most common malignancy globally (1, 2). Although the overall prognosis of thyroid cancer is favorable, lymph node metastasis (LNM) is relatively common among patients, with an incidence ranging from 20% to 50%. It increases the risk of death by 46% and is closely associated with higher recurrence rates, reduced overall survival, and impaired quality of life (3–5). Therefore, accurate preoperative assessment of LNM is essential for guiding surgical strategies.

Diabetes, as the most prevalent metabolic disorder, affects over 537 million individuals worldwide. In recent years, it has been increasingly recognized as a potential risk factor for various malignancies, including thyroid cancer (6, 7). A growing body of evidence suggests that diabetes not only increases the risk of cancer development but may also influence the biological behavior and prognosis of the disease. A 10-year prospective study reported that diabetic patients have a 25% higher risk of developing thyroid cancer, and those with a history of diabetes and elevated fasting blood glucose have a significantly increased risk of thyroid cancer (8–10). Furthermore, diabetic patients are more susceptible to LNM, which may lead to poorer outcomes (11, 12). With the concurrent rise in the incidence of diabetes and thyroid cancer, the intersection of these two conditions warrants particular attention. Notably, type 2 diabetes accounts for about 95% of all diabetes cases, and patients with thyroid cancer complicated by type 2 diabetes may represent a subgroup with distinct biological characteristics and potentially worse clinical outcomes. Thus, more accurate preoperative risk assessment for this subgroup is especially important.

Contrast-enhanced ultrasound (CEUS) is an emerging ultrasound (US) imaging technique that enables real-time evaluation of vascular perfusion in focal lesions. Its primary advantage lies in the precise assessment of the sequence and intensity of tumor perfusion and blood flow distribution, which are characteristic features of malignant tumors (13). In recent years, several studies have evaluated the utility of CEUS in thyroid US examinations. For example, the authors of the 2017 guidelines from the European Federation of Societies for Ultrasound in Medicine and Biology (EFSUMB) indicated that CEUS can be applied for the differential diagnosis of focal thyroid lesions (14). Compared with conventional US, CEUS may enhance the diagnosis of thyroid cancer and has potential value in predicting LNM (15). Currently, relatively few studies have investigated the prediction of LNM in thyroid cancer using dynamic CEUS. As a progressively adopted technique, prediction of LNM based on CEUS represents a promising research direction.

Radiomics, a widely applied research approach in recent years, extracts high-throughput quantitative features from medical images (16). Several radiomics studies have not only demonstrated the feasibility of predicting LNM in thyroid cancer patients but also combined US radiomics features with clinical data to construct robust predictive models (17–19). However, most of these studies focus only on the tumor itself and do not fully consider the role of the tumor microenvironment, despite increasing evidence suggesting that the peri-tumoral region plays a critical role in tumor progression and metastasis (20). Dynamic CEUS can provide multi-temporal tumor features and offers distinct advantages in evaluating intra-tumoral and peri-tumoral blood flow. The integration of CEUS with intra-tumoral and peri-tumoral radiomics may further improve predictive performance.

Against this background, this study aimed to establish a model for predicting LNM in thyroid cancer patients with diabetes based on radiomics features extracted from dynamic CEUS. By integrating multi-temporal perfusion features with intra-tumoral and peri-tumoral radiomics analysis, a non-invasive and accurate preoperative assessment tool for clinical application is expected, which may optimize surgical decision-making and promote individualized management of high-risk diabetic populations.

## 2 Materials and methods

### 2.1 Patients

**Figure 1:**
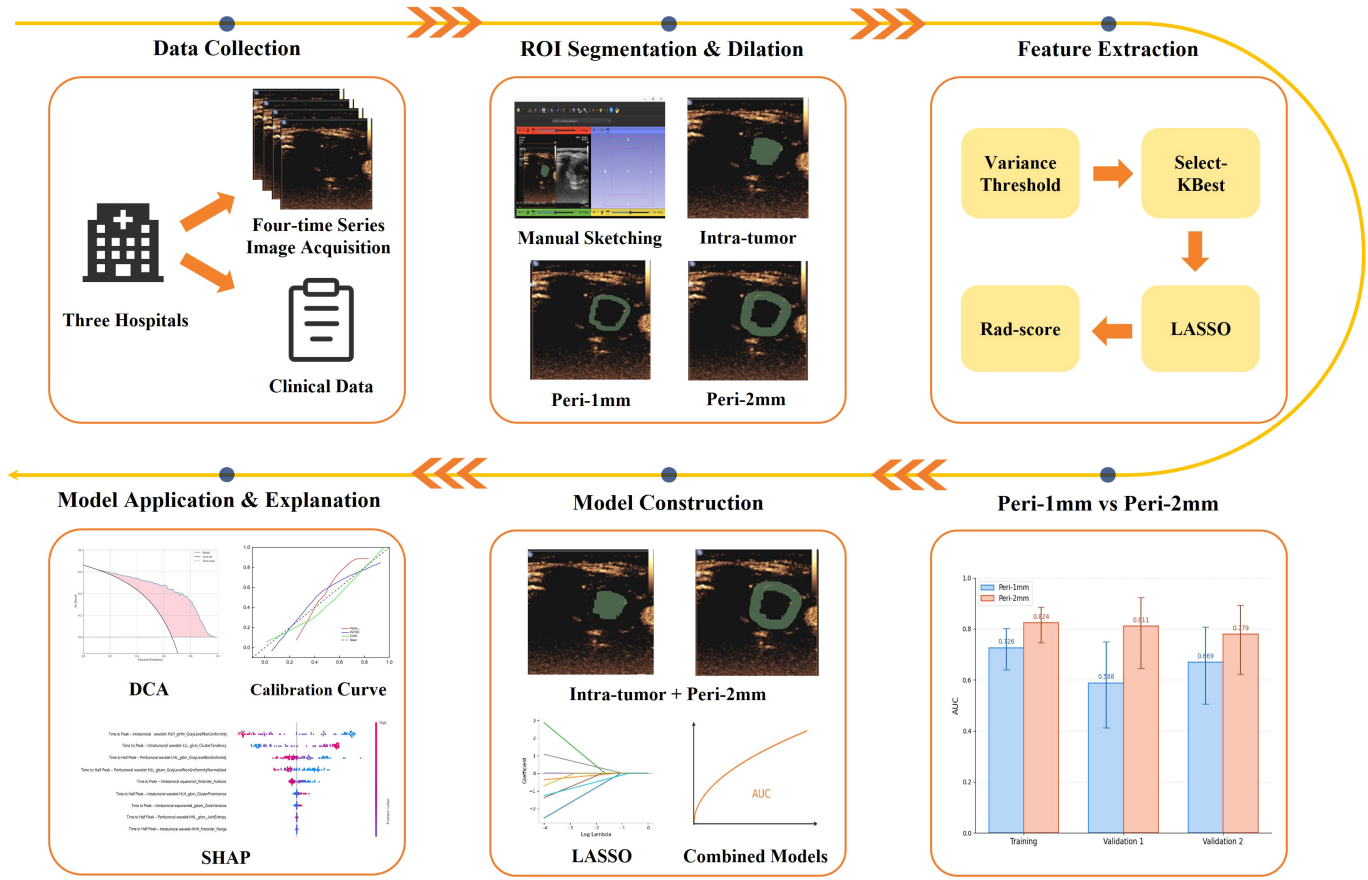
Schematic overview of the study workflow.

This retrospective study was approved by the Ethics Committee of our hospital in accordance with the Declaration of Helsinki. Due to data anonymization, the requirement for patients to provide written informed consent was waived. Data were retrospectively collected from thyroid cancer patients treated at Center 1 (n=126) from June 2020 to March 2025, as well as from patients treated at Center 2 (n=36) and Center 3 (n=41) during the same period. The inclusion criteria were as follows (1): patients with pathologically confirmed thyroid cancer who underwent initial surgical treatment (2); performance of dynamic CEUS within 1 month before surgery (3); no prior treatment history for thyroid-related diseases (4); clinically confirmed diabetes. The exclusion criteria were as follows: (1) presence of malignant tumors in other organs; (2) history of thyroid or cervical lymph node surgery, or acute/chronic inflammation; (3) unclear imaging data or incomplete clinical information; (4) severe cardiac, hepatic, or renal dysfunction. Patients from Center 1 were assigned to the training cohort, whereas those from Center 2 and Center 3 served as external validation cohort 1 and external validation cohort 2, respectively. The study flow chart is shown in **Figure 1**. The clinical data included age, gender, maximum grayscale diameter, BRAF mutation status, tumor location, presence of multiple tumors, and US features of thyroid cancers. The baseline characteristics are presented in **Table 1**.

**Table 1:** Baseline clinical characteristics of patients in the training and validation groups.

Variables	Total (n = 203)	Training group (n = 126)	External validation group 1 (n = 36)	External validation group 2 (n = 41)	Statistic	*P*
Age, Mean ± SD	44.73 ± 10.83	44.14 ± 10.63	46.75 ± 11.37	44.76 ± 10.99	F=0.81	0.446
Grayscale maximum diameter, Mean ± SD	0.90 ± 0.45	0.92 ± 0.45	0.92 ± 0.52	0.83 ± 0.37	F=0.65	0.526
BRAF, n (%)					χ²=0.00	0.998
Negative	50 (24.63)	31 (24.60)	9 (25.00)	10 (24.39)		
Positive	153 (75.37)	95 (75.40)	27 (75.00)	31 (75.61)		
Gender, n (%)					χ²=1.33	0.514
Female	160 (78.82)	97 (76.98)	28 (77.78)	35 (85.37)		
Male	43 (21.18)	29 (23.02)	8 (22.22)	6 (14.63)		
Tumor location, n (%)					χ²=6.07	0.416
Isthmus	9 (4.43)	6 (4.76)	2 (5.56)	1 (2.44)		
Upper	49 (24.14)	32 (25.40)	6 (16.67)	11 (26.83)		
Mid	89 (43.84)	57 (45.24)	19 (52.78)	13 (31.71)		
Lower	56 (27.59)	31 (24.60)	9 (25.00)	16 (39.02)		
Multiplicity, n (%)					χ²=0.33	0.846
No	117 (57.64)	74 (58.73)	21 (58.33)	22 (53.66)		
Yes	86 (42.36)	52 (41.27)	15 (41.67)	19 (46.34)		
Tumor boundary, n (%)					-	1.000
Clear	24 (11.82)	15 (11.90)	4 (11.11)	5 (12.20)		
Unclear	179 (88.18)	111 (88.10)	32 (88.89)	36 (87.80)		
Shape, n (%)					χ²=0.32	0.852
Regular	34 (16.75)	21 (16.67)	7 (19.44)	6 (14.63)		
Irregular	169 (83.25)	105 (83.33)	29 (80.56)	35 (85.37)		
Aspect ratio, n (%)					χ²=0.29	0.864
<1	105 (51.72)	67 (53.17)	18 (50.00)	20 (48.78)		
≥1	98 (48.28)	59 (46.83)	18 (50.00)	21 (51.22)		
Internal echogenicity, n (%)					χ²=1.52	0.467
Low echo	158 (77.83)	100 (79.37)	29 (80.56)	29 (70.73)		
Extremely low echo	45 (22.17)	26 (20.63)	7 (19.44)	12 (29.27)		
Vascularity, n (%)					χ²=0.55	0.759
Absence	119 (58.62)	73 (57.94)	20 (55.56)	26 (63.41)		
Presence	84 (41.38)	53 (42.06)	16 (44.44)	15 (36.59)		
Macrocalcification, n (%)					-	0.939
Absence	188 (92.61)	117 (92.86)	34 (94.44)	37 (90.24)		
Patchy	8 (3.94)	5 (3.97)	1 (2.78)	2 (4.88)		
Punctate	7 (3.45)	4 (3.17)	1 (2.78)	2 (4.88)		
Two-dimensional lymph node, n (%)					χ²=1.96	0.375
Not explored	156 (76.85)	97 (76.98)	25 (69.44)	34 (82.93)		
Enlargement	47 (23.15)	29 (23.02)	11 (30.56)	7 (17.07)		
Two-dimensional capsular invasion, n (%)					χ²=1.06	0.590
Non infringement	172 (84.73)	107 (84.92)	32 (88.89)	33 (80.49)		
Infringement	31 (15.27)	19 (15.08)	4 (11.11)	8 (19.51)		

SD, standard deviation

### 2.2 CEUS scan

All ultrasound and CEUS examinations were performed using a Resona 7S ultrasound system (Mindray, Shenzhen, China). Conventional grayscale ultrasound was initially conducted using a high-frequency linear transducer (L14-5WU, 5–14 MHz) to evaluate the thyroid lesion and surrounding cervical region. The lesion was scanned in multiple planes, and the section showing the maximum tumor diameter was selected as the target plane for subsequent CEUS acquisition. CEUS was then performed in contrast-specific imaging mode using a linear transducer (L9-3, 3–9 MHz). The mechanical index was set to 0.10, and the frame rate was maintained at 10 frames per second. During the examination, patients were placed in the supine position with the neck slightly extended. They were instructed to breathe quietly and avoid swallowing or speaking during image acquisition to minimize motion artifacts. After stabilization of the target plane, 2.4 mL of SonoVue contrast agent (Bracco, Italy) was administered as an intravenous bolus through the antecubital vein, followed immediately by a 5-mL normal saline flush. Continuous dynamic CEUS cine loops were recorded immediately after contrast injection. The entire enhancement and washout process of the lesion was continuously observed and stored in DICOM format for subsequent radiomics analysis. Four representative frames corresponding to predefined dynamic CEUS phases were extracted from the cine loops: time to enhancement (TTE), defined as the first visible enhancement within the tumor; time to half-peak (TTHP), defined as the time point at which the enhancement intensity reached about half of the peak intensity; time to peak (TTP), defined as the frame showing maximal enhancement intensity of the tumor; and washout time (WT), defined as the time point at which the enhancement of the lesion began to decrease after peak enhancement. These four time points were subsequently used for intra-tumoral and peri-tumoral ROI segmentation and radiomics feature extraction.

### 2.3 ROI segmentation, processing, and feature extraction

Histogram equalization and image standardization were performed on the CEUS images. A radiologist with 5 years of experience (Radiologist 1) manually delineated the region of interest (ROI) within the thyroid cancer tumor on the US images at the four time points using the 3D segmentation software 3D-Slicer (Version 4.8.0). For the peri-tumoral ROI in the CEUS images, morphological dilation operations based on the 3D segmentation software were applied with radii of 1.0 mm and 2.0 mm to obtain the corresponding ROIs. The selection of 1 mm and 2 mm was based on prior radiomics evidence in thyroid cancer and biological considerations of the tumor microenvironment. Previous studies have consistently validated that a 1–2 mm peritumoral margin provides optimal predictive performance for ultrasound-based radiomics models (21–23), while margins exceeding 2 mm tend to introduce non-specific parenchymal signals, increase feature redundancy, and reduce model specificity. Furthermore, larger peritumoral regions (≥3 mm) would exacerbate the partial volume effect in dynamic CEUS, leading to non-specific perfusion signals from normal thyroid parenchyma and compromised feature reliability. Using the open-source Pyradiomics toolkit, radiomics features were extracted from the intra-tumoral ROI and the peri-tumoral ROI of the CEUS images, respectively. The extracted hand-crafted features were classified into the following groups: shape; first-order; grey-level co-occurrence matrix (GLCM); grey-level size zone matrix (GLSZM); grey-level run length matrix (GLRLM); neighborhood grey-tone difference matrix (NGTDM); and grey-level correlative matrix.

### 2.4 Feature selection

Feature selection was conducted only in the training cohort to prevent information leakage. Radiologist 1 repeated the ROI segmentation after 2 weeks, and the intra-class correlation coefficient (ICC) was assessed in 30 randomly selected patients. In addition, another radiologist with 10 years of experience (Radiologist 2) independently performed ROI segmentation for ICC evaluation. Radiomics features with an ICC value > 0.8 were retained. Variance Threshold, SelectKBest, and LASSO algorithms were sequentially applied for dimensionality reduction. First, low-variance features were eliminated using the Variance Threshold method with a threshold of 0.8 as an initial filtering step. Next, univariate feature screening was conducted using SelectKBest based on analysis of variance (ANOVA), and features significantly associated with LNM in the training cohort were retained. Finally, LASSO regression was applied to further reduce feature dimensionality and identify the most informative features. The regularization parameter λ was determined by cross-validation in the training cohort.

### 2.5 Comparison of peri-tumoral regions

For the radiomics features extracted from the 1 mm and 2 mm peri-tumoral regions through the three-step process described above, radiomics scores were calculated using the features and their coefficients selected in the final LASSO step. The formula used to calculate the relevant radiomics scores is provided in the **Supplementary Materials**. The area under the receiver operating characteristic curve (AUC) values of the radiomics scores between the two peri-tumoral regions were compared across the three cohorts to determine select the optimal peri-tumoral region.

### 2.6 Model establishment

Similarly, a radiomics score model was constructed for the intra-tumoral region using LASSO to obtain the intra-tumoral radiomics score. This score, together with the radiomics score of the optimal peri-tumoral region obtained in the previous step, was combined with clinical baseline information for logistic regression analysis. For each parameter, the odds ratio (OR) and its corresponding 95% confidence interval (95% CI) were calculated. Subsequently, variables with a P-value less than 0.05 were selected for inclusion in the multivariate logistic regression model. The final logistic regression model included all significant parameters identified through this process.

### 2.7 Statistical analysis

SPSS 23.0 for Windows and R software (Version 4.2.3) were used for statistical analysis in this study. Variables conforming to a normal distribution were expressed as mean ± standard deviation, whereas non-normally distributed data were presented as median and interquartile range (IQR). For the comparison of continuous variables between groups, one-way ANOVA was used for normally distributed data, and the Kruskal-Wallis test was applied for non-normally distributed data. The chi-square test was used to analyze differences in categorical variables. A two-tailed P-value < 0.05 was considered statistically significant. Univariate and multivariate logistic regression analyses were performed in this study. Factors with a P-value < 0.05 in the univariate analysis were included in the multivariate model. The discriminative ability of the established models was determined using the area under the receiver operating characteristic curve (AUC). Calibration curves were plotted to assess the agreement between predicted probabilities and observed outcomes. Validation was performed using 1,000 bootstrap resamples to evaluate the robustness and stability of the model. Decision curve analysis (DCA) was also conducted to quantify the net benefit and assess the clinical utility of the model across a range of threshold probabilities.

To assess the added value of the radiomics model, two baseline models were constructed for comparison: a clinical baseline incorporating Age, Gender, Tumor size, BRAF mutation, Tumor multiplicity, and Capsular invasion, and a conventional ultrasound baseline using Tumor shape, Margin, Echogenicity, Macrocalcification, Vascularity, and Lymph node enlargement on B−mode imaging. In univariate analysis, none of these clinical or US variables were significantly associated with lymph node metastasis (all P > 0.05), indicating that the two baseline models’ limited performance. Both models were developed through multivariate logistic regression in the training cohort and subsequently validated across the same independent cohorts. All variables were directly entered into the model without further feature selection or manual exclusion. To further validate the predictive value of routine clinical and ultrasound features, three machine learning models (random forest, support vector machine, and XGBoost) were constructed using the same variables. All models were trained in the training cohort with 5−fold cross−validation for default parameter optimization and tested in the external validation cohorts.

## 3 Results

### 3.1 Baseline characteristics

Among the 203 participants, 126 (62.1%) were assigned to the training group, 36 (17.7%) to the external validation group 1, and 41 (20.2%) to the external validation group 2. There were no significant differences in the distribution of LNM status among the study groups (all P > 0.05). Specifically, the proportions for absence of LNM were 53.9%, 47.2% and 51.2% in each group, while for presence, they were 46.0%, 52.8% and 48.8%, respectively. The mean age of the participants was 44.7 ± 10.8 years, and 78.8% were female. The baseline characteristics were comparable across the three groups. No significant inter-group differences were observed in any baseline data (all P > 0.05).

### 3.2 Radiomics feature selection

**Figure 2:**
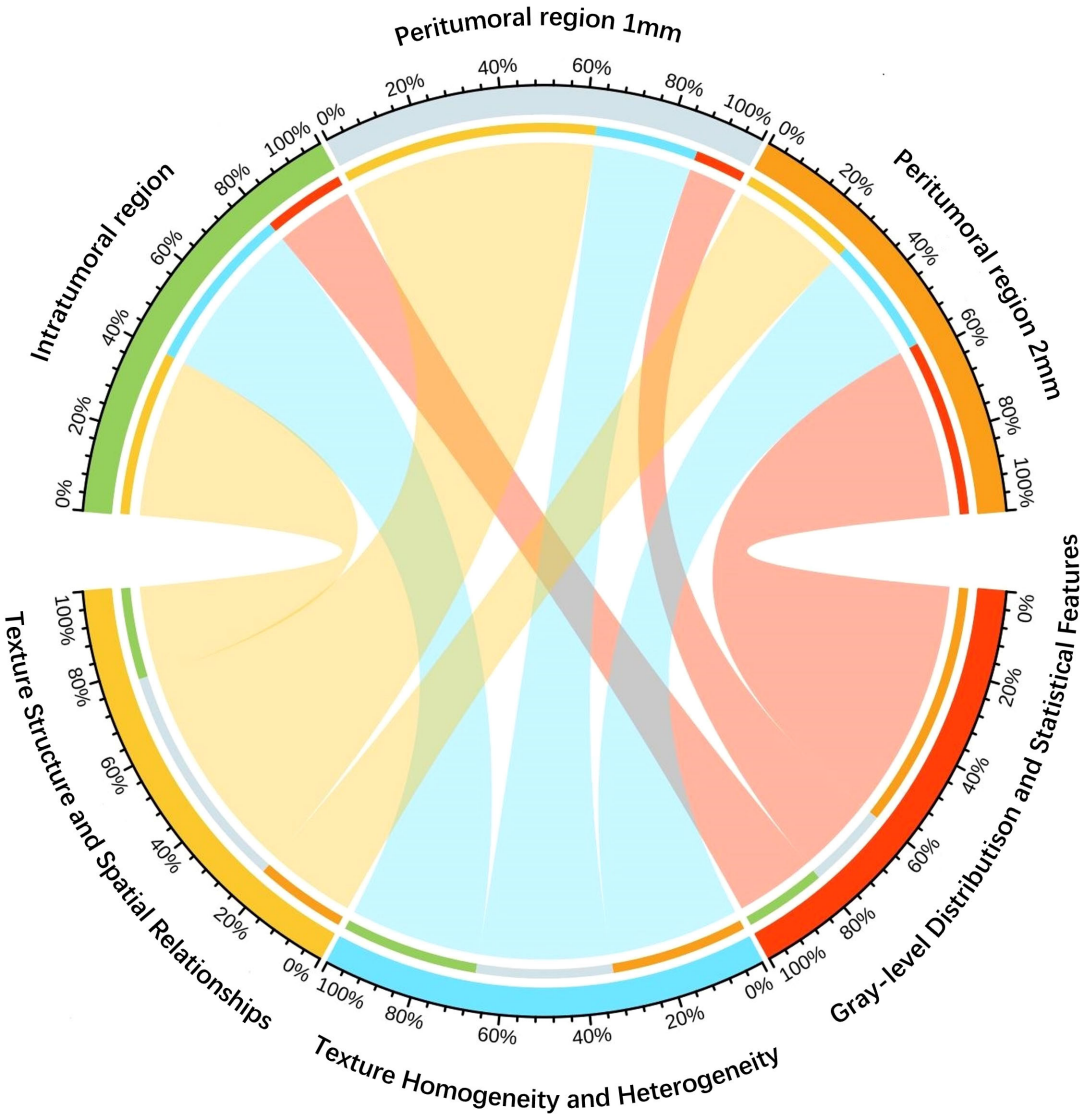
Chord diagram of associations between tumor regions and radiomics feature categories.

A total of 1409 radiomics features were extracted for each time phase of CEUS in each patient. After the three-step feature selection process, 7 features were retained for the intra-tumoral region, 8 features for the 1 mm peri-tumoral region, and 7 features for the 2 mm peri-tumoral region. These 22 features were categorized into three groups based on their physical meanings: gray-level distribution and statistical features, texture uniformity and heterogeneity features, and texture structure and spatial relationship features. The detailed classification results are provided in the **Supplementary Materials**. The associations among the intra-tumoral region, the 1 mm and 2 mm peri-tumoral regions, and their corresponding radiomics features were further analyzed. A chord diagram was used to visually represent the quantitative relationships between each region, where the thickness of the connecting lines indicates the strength of the association (**Figure 2**). It was observed that the intra-tumoral region was mostly associated with texture homogeneity and heterogeneity features, the 1mm peri-tumoral region was primarily related to texture structure and spatial relationship features, and the 2 mm peri-tumoral region was largely correlated with grey-level distribution and statistical features.

### 3.3 Determination of the optimal peri-tumoral region

Based on the radiomics scores established for the two peri-tumoral regions (1 mm vs 2 mm), the 2 mm peri-tumoral region showed the highest AUC in the training cohort and maintained a consistently higher AUC than the 1 mm region in both external validation cohorts, with AUC values as high as 0.824 (95% CI: 0.746–0.886), 0.811 (95% CI: 0.646–0.922), and 0.779 (95% CI: 0.622–0.893), respectively. The detailed classification results are presented in **Table 2**. The detailed calculation formula for the radiomics score is provided in the **Supplementary Materials**.

**Table 2:** The DeLong test employed to compare the performance between the 1mm and 2mm peri-tumoral region.

Variables	Training group	External validation group 1	External validation group 2
Peri-1mm vs Peri-2mm			
Difference between areas	0.981	0.223	0.110
95% Confidence Interval	0.0674-0.129	-0.0481–0.494	-0.114-0.333
Standard Error	0.0157	0.138	0.114
z statistic	6.267	1.612	0.959
Significance level	< 0.0001	0.1070	0.3375

### 3.4 Construction of the final radiomics prediction model

**Figure 3:**
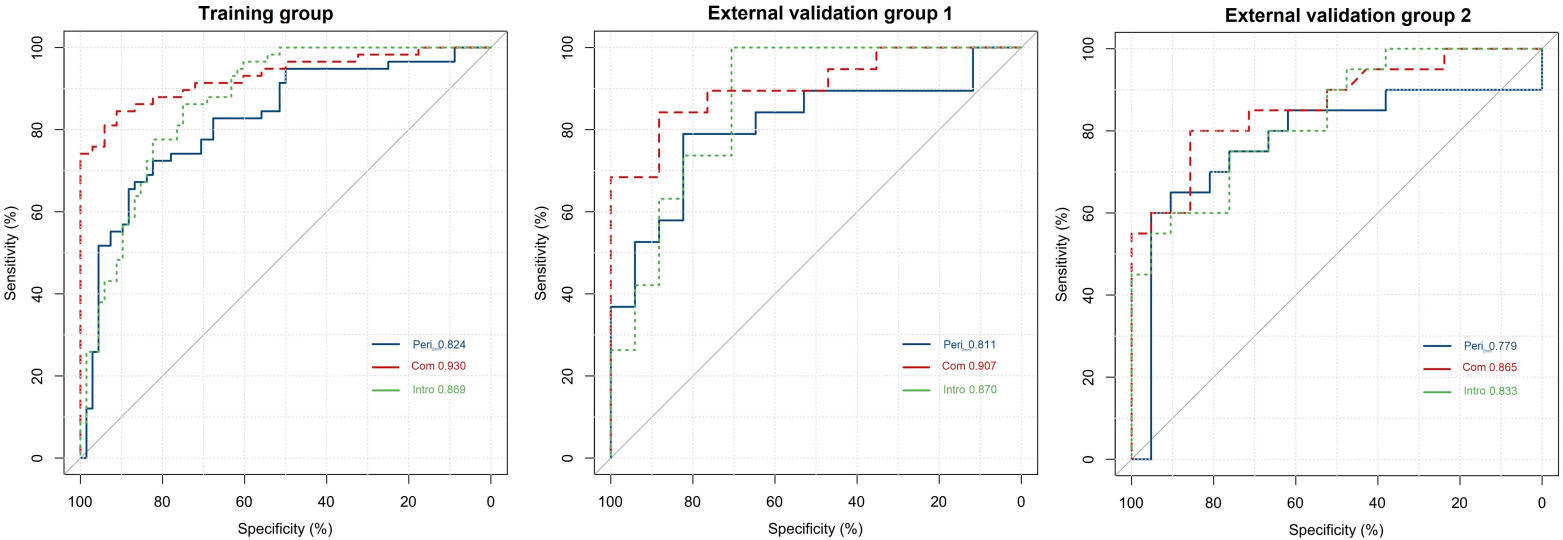
ROC curves comparing predictive performance of radiomics models across cohorts.

**Figure 4:**
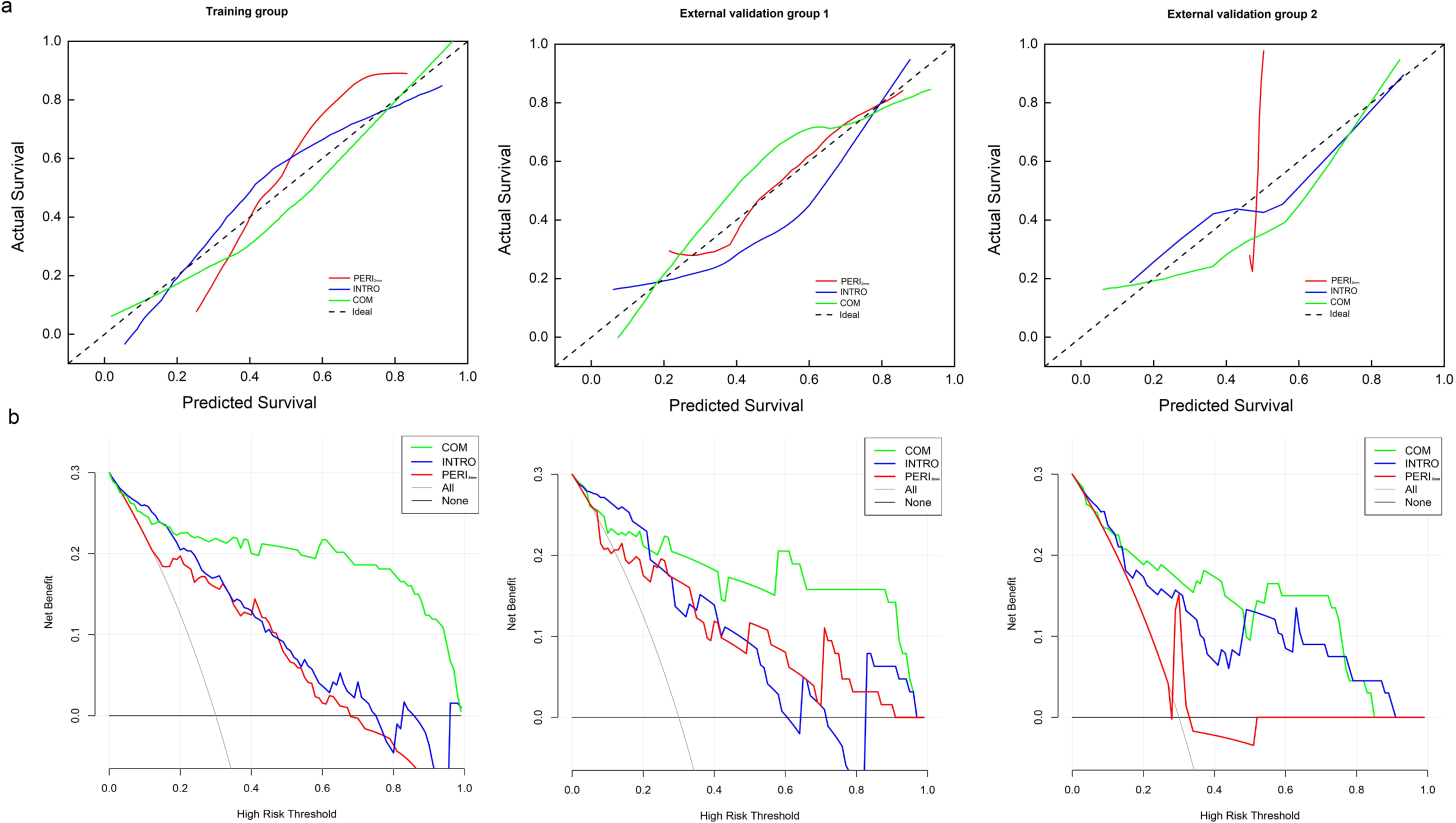
Calibration curves and decision curve analysis (DCA) of the combined radiomics model. The upper row shows calibration plots of predicted versus actual lymph node metastasis probability in the training cohort, external validation cohort 1 and external validation cohort 2, respectively **(a)**. The lower row presents DCA evaluating the net clinical benefit of the model across different risk thresholds in the three cohorts. Three models (COM, INTRO, PERI) as well as the reference lines of "All" and "None" are displayed **(b)**.

Through univariate logistic regression analysis, none of the clinical and conventional US indicators showed statistically significant associations. Consequently, the final radiomics model was constructed using multivariate logistic regression, integrating the intratumoral radiomics score from CEUS images and the 2 mm peritumoral radiomics score from CEUS images (**Table 3**). Across the three cohorts, the final model achieved higher AUC values than the intratumoral-only model (training cohort: 0.930, external validation cohort 1: 0.907, external validation cohort 2: 0.865), indicating the model’s favorable applicability across multiple cohorts (**Figure 3**). The calibration curves (**Figure 4a**) illustrate the LNM prediction performance of the final model across different populations. In all subgraphs, the horizontal axis represents the “Predicted LNM Probability” ranging from 0.0 to 1.0, and the vertical axis corresponds to the actual LNM status of the samples in each cohort. From the overall distribution, in the Training group, there was good agreement between the predicted values and LNM status, and the data points were reasonably distributed along the theoretical fitting trend. In the two external validation groups, although the data dispersion was slightly greater than that in the Training group, the predicted LNM probability still reflected the metastasis status of the samples to a certain extent, demonstrating the model’s predictive stability across independent populations. **Figure 4b** presents the comparison of the net benefit of the combined model under different intervention strategies. The vertical axis represents the net benefit value, where a higher value indicates a greater clinical benefit provided by the model under that strategy, and the horizontal axis represents the decision-making threshold. The analysis showed that when the final model strategy was applied, the net benefit value of the model was higher than that of other strategies, within most of the threshold range.

**Table 3:** Logistic regression analysis results.

Variables	Univariate logistic regression					Multivariate logistic regression				
	β	S.E	Z	*P*	OR (95%CI)	β	S.E	Z	*P*	OR (95%CI)
BRAF (Negative 0, Positive 1), n (%)										
0					1.00 (Reference)					
1	0.05	0.42	0.11	0.911	1.05 (0.46 ~ 2.36)					
Gender (Male 1, Female 0), n (%)										
0					1.00 (Reference)					1.00 (Reference)
1	1.24	0.45	2.74	0.006	3.45 (1.42 ~ 8.37)	1.29	0.56	2.28	0.078	3.62 (1.20 ~ 10.94)
Tumor location, n (%)										
Isthmus					1.00 (Reference)					
Upper	-0.82	0.94	-0.87	0.382	0.44 (0.07 ~ 2.76)					
Mid	-0.87	0.91	-0.96	0.337	0.42 (0.07 ~ 2.48)					
Lower	-1.02	0.94	-1.08	0.278	0.36 (0.06 ~ 2.28)					
Tumor boundary, n (%)										
Clear					1.00 (Reference)					
Unclear	-0.03	0.55	-0.05	0.958	0.97 (0.33 ~ 2.86)					
Shape, n (%)										
Regular					1.00 (Reference)					
Irregular	0.39	0.49	0.80	0.426	1.48 (0.57 ~ 3.86)					
Aspect ratio, n (%)										
<1					1.00 (Reference)					
≥1	0.37	0.36	1.02	0.309	1.44 (0.71 ~ 2.91)					
Internal echogenicity, n (%)										
Low echo					1.00 (Reference)					
Extremely low echo	-0.39	0.45	-0.87	0.386	0.68 (0.28 ~ 1.64)					
Vascularity, n (%)										
Absence					1.00 (Reference)					
Presence	0.34	0.36	0.94	0.347	1.41 (0.69 ~ 2.86)					
Macrocalcification, n (%)										
Absence					1.00 (Reference)					
Patchy	-1.23	1.13	-1.09	0.277	0.29 (0.03 ~ 2.69)					
Punctate	1.25	1.17	1.07	0.284	3.50 (0.35 ~ 34.64)					
Two-dimensional lymph node, n (%)										
Not explored					1.00 (Reference)					
Enlargement	0.85	0.43	1.95	0.051	2.33 (0.99 ~ 5.47)					
Two-dimensional capsular, n (%)										
Non infringement					1.00 (Reference)					
Infringement	0.82	0.51	1.60	0.110	2.27 (0.83 ~ 6.23)					
Age, Mean ± SD	0.00	0.02	0.16	0.870	1.00 (0.97 ~ 1.04)					
Grayscale maximum diameter, Mean ± SD	0.45	0.41	1.09	0.275	1.56 (0.70 ~ 3.48)					
Intratumoral LASSO results	0.07	0.01	5.56	<.001	1.07 (1.04 ~ 1.09)	0.07	0.01	5.42	<.001	1.07 (1.04 ~ 1.10)
Peri-2mm tumoral LASSO results	0.05	0.01	2.81	0.047	0.99 (0.97 ~ 1.01)	0.05	0.01	2.61	0.032	1.01 (1.01 ~ 1.07)

OR, Odds Ratio; CI, Confidence Interval; SD, standard deviation.

The clinical and conventional US baseline models yielded poor AUC values (0.62–0.67) across all cohorts, which was consistent with the non-significant effects of clinical and US features in univariate analysis. The machine learning baseline models achieved AUC values of 0.67–0.73 across cohorts, which remained modest and comparable to the logistic regression baseline models. In contrast, the combined radiomics model showed significantly better performance, demonstrating its clear incremental value (**Supplementary Materials**).

### 3.5 Model interpretability

**Figure 5:**
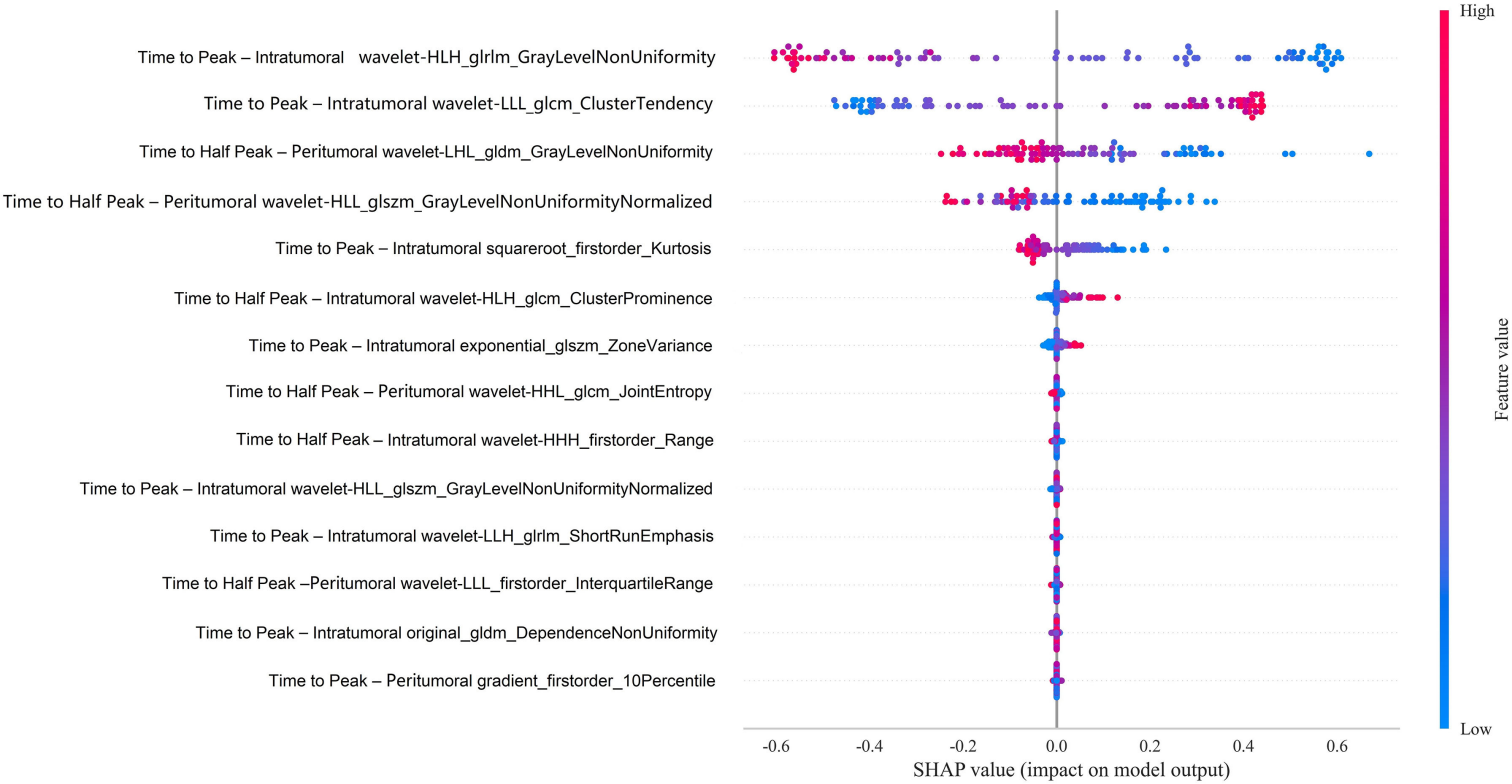
SHAP summary plot for feature interpretability of the combined radiomics model: the plot employs a color gradient ranging from blue to red, and the values on the horizontal axis increase from left to right, with a range of -0.6 to 0.6. A larger ab-solute value of the negative value on the horizontal axis indicates a higher probability of a negative prediction result; similarly, a larger absolute value of the positive value corresponds to a higher probability of a positive result.

Seven radiomics features in the 2-mm peritumoral region played significant role in predicting LNM in thyroid cancer. According to their imaging characteristics, these features were classified into three groups: gray-level distribution and statistical features, texture heterogeneity features, and texture structure/spatial relationship features. Collectively, they reflect signal intensity variation, regional non-uniformity, and spatial complexity in the peritumoral region, suggesting that a more heterogeneous peritumoral imaging pattern may be associated with a higher likelihood of LNM. To visualize the contribution of intratumoral and 2mm peritumoral radiomics to the final logistic regression model, SHAP (SHapley Additive exPlanations) plots (**Figure 5**) were used to demonstrate the contribution of each radiomics feature to the prediction. Taking the “Time to Peak – Intratumoral wavelet - HLH_glszm_GrayLevelNonUniformity” feature as an example, this feature contributed most substantially to the combined model. An increase in this value significantly reduced the probability of LNM. In contrast, the “Time to Half Peak – Peritumoral wavelet - LHL_gldm_GrayLevelNonUniformityNormalized” feature ranked third in contribution, and an increase in this value reduced the probability of lymph node metastasis.

## 4 Discussion

Surgical resection remains the primary treatment modality for thyroid cancer, and the formulation of surgical strategies depends on accurate preoperative prediction of LNM, particularly for patients with type 2 diabetes who exhibit distinct tumor microenvironmental alterations and a higher risk of LNM (24, 25). The biological basis of imaging features for LNM prediction in this specific population has not been fully clarified, and the value of peri-tumoral radiomics features based on dynamic CEUS remains under investigation (26). In this study, a combined radiomics model based on intra-tumoral and 2 mm peri-tumoral features from dynamic CEUS was constructed, and it was found that the TTP and TTHP phases were key time points for extracting effective features, while the 2 mm peri-tumoral region significantly improved the predictive performance of the model for LNM.

Compared with non−diabetic patients, individuals with type 2 diabetes exhibit chronic hyperglycemia, hyperinsulinemia, elevated IGF−1, oxidative stress, and systemic low−grade inflammation, all of which promote tumor angiogenesis, invasion, and lymph node metastasis. These metabolic and microenvironmental alterations result in more aggressive tumor behavior and a higher risk of LNM that cannot be accurately estimated using conventional models developed for the general population. A substantial body of research has shown that diabetic patients present various abnormal indicators, including altered secretion of thyroid-stimulating hormone (TSH), oxidative stress damage, hyperinsulinemia, and elevated levels of IGF-1. For example, plasma TSH concentration is closely associated with the occurrence and progression of thyroid cancer, and elevated TSH levels promote tumor cell proliferation, invasion, and metastasis (27). In diabetic patients, levels of reactive oxygen species (ROS) and oxidative stress are increased, which affect cell growth and proliferation, leading to DNA mutations and potentially contributing to multistage carcinogenesis (28). Elevated plasma IGF-1 levels can significantly promote the proliferation of thyroid cells and the migration of tumor cells. The underlying mechanism may be related to the activation of insulin and IGF-1 receptors, as well as downstream PI3K/PKB signaling pathways, which inhibit the activity of the tumor suppressor factor FoxO3a (29, 30). Therefore, identifying of reliable biomarkers for accurate preoperative prediction of LNM in the diabetic population is of considerable importance.

CEUS has been widely applied, particularly for differentiating benign and malignant lesions. Li et al. (31) conducted a prospective study involving eight major centers to evaluate the application of CEUS in assessing the malignancy of thyroid nodules. The results showed that dynamic CEUS combined with conventional US could effectively distinguish the ACR-TIRADS classification of thyroid nodules. Ren et al. (32) constructed a model for distinguishing benign from malignant thyroid nodules classified as ACR TI-RADS 4–5 using a dual-modal radiomics nomogram based on CEUS. Compared with ACR TI-RADS, the rate of unnecessary Fine-Needle Aspiration Biopsy (FNAB) was significantly reduced. Chen et al. (33) classified and predicted the benignity and malignancy of thyroid nodules categorized as Category 4 in the Chinese Thyroid Imaging Reporting and Data System (C-TIRADS) by combining 2D-US with five key frames of CEUS. The Random Forest (RF) model integrating 2D-US and CEUS key frames achieved performance comparable to that of senior radiologists in the test cohort. More recently, CEUS has also been applied to the evaluation of LNM. For example, Jiang et al. (34) employed CEUS combined with a clinical radiomics nomogram to predict LNM in thyroid cancer. Their results demonstrated that the clinical radiomics nomogram exhibited good performance in both the training set (AUC = 0.820) and the validation set (AUC = 0.814), and DCA indicated that the clinical radiomics nomogram had good clinical utility. Chen et al. (35) explored the role of CEUS in risk stratification for LNM in thyroid cancer. The study revealed that the nomogram had higher predictive value for LNM than US or CEUS features alone. With a Nomo score of 0.428 as the cut-off value, it showed good performance in stratifying high-risk and low-risk groups. However, previous studies have neither considered the potential value of the peritumoral region nor incorporated the temporal information derived from CEUS imaging. In the present study, a CEUS-based radiomics model using intratumoral and 2mm peritumoral regions was constructed and validated, with analyses conducted and validated across four-time phases. It was demonstrated that combining intratumoral and peritumoral radiomics features has the potential to non-invasively capture biological processes associated with metastasis. Notably, among the fourteen extracted features, eight were TTP features and the remainder were TTHP features, suggesting that these time points may best reflect differences in the intratumoral and peritumoral microenvironments of thyroid cancer.

The TTP phase represents the peak perfusion stage of the contrast agent in tumor and peri-tumoral microcirculation, and the imaging features at this stage can most intuitively reflect the status of tumor angiogenesis, the core biological basis of tumor invasion and metastasis. For type 2 diabetic patients, hyperinsulinemia, elevated IGF-1 levels and chronic oxidative stress can activate the PI3K/Akt/FoxO3a signaling pathway, which not only promotes abnormal proliferation of tumor blood vessels but also induces microvascular endothelial damage and increased vascular permeability in the peri-tumoral region. These changes may result in significant heterogeneity of contrast agent perfusion in the intra-tumoral and peri-tumoral regions at the TTP phase, which can be quantified by radiomics features such as GLSZM_GrayLevelNonUniformity (intra-tumoral) and Firstorder_Range (peri-tumoral). The SHAP analysis further confirmed that the TTP phase intra-tumoral texture heterogeneity feature was the most contributory factor to the model, which is consistent with the biological principle that abnormal angiogenesis is a prerequisite for thyroid cancer cell invasion and lymph node metastasis. In patients with type 2 diabetes, chronic low-grade inflammation and glycation end products may cause fibrous deposition, immune cell infiltration and microvascular basement membrane thickening in the peri-tumoral region of thyroid cancer, which may disrupt the integrity of the peri-tumoral tissue barrier and alter the clearance rate of the contrast agent. The TTHP phase features (such as GLDM_GrayLevelNonUniformityNormalized in the peri-tumoral region) may capture these structural and functional abnormalities, and SHAP analysis showed that an increase in this feature value was a protective factor for LNM— this may be because a higher normalized gray level non-uniformity indicates a more regular peri-tumoral microvascular structure and an intact tissue barrier, which can inhibit tumor cell invasion into the lymphatic system.

Seven radiomics features in the 2mm peritumoral region were finally selected. Based on their physical characteristics, these seven features can be categorized into three types: gray-level distribution and statistical features (Wavelet- HHH_firstorder_Range,Wavelet-LLL_firstorder_InterquartileRange, Gradient_firstorder_10Percentile), texture uniformity and heterogeneity features (Wavelet-LHL_gldm_GrayLevelNonUniformity, Wavelet- HLL_glszm_GrayLevelNonUniformityNormalized), and texture structure and spatial relationship features (Wavelet-HLH_glcm_ClusterProminence, Wavelet-HHL_glcm_JointEntropy). Gray-level distribution and statistical features may reflect the overall distribution of gray-level values in the peritumoral region and their degree of dispersion. For example, the gray-level range and interquartile range may reflect fluctuations in local signal intensity, while low-percentile gray-level values may indicate the presence of tissue with low density or reduced metabolic activity in the peritumoral region. In the context of diabetes, chronic inflammation and microvascular lesions can alter blood supply and metabolism in the peritumoral region of thyroid cancer, leading to greater fluctuations in signal intensity on imaging. This variation is closely associated with the invasive and metastatic potential of the tumor, suggesting that diabetes may promote LNM by increasing instability in peritumoral gray-level statistical features. Texture uniformity and heterogeneity features primarily describe whether the gray-level or regional distribution in the peritumoral tissue is uniform. A higher degree of non-uniformity may indicate stronger heterogeneity and more complex tissue composition. The peritumoral region of thyroid cancer in diabetic patients is often accompanied by chronic inflammatory responses, fibrous deposition, and immune cell infiltration. These pathological changes may lead to marked gray-level inhomogeneity in imaging. A high degree of non-uniformity reflects disorder and instability in the peritumoral microenvironment, which not only facilitates tumor cell invasion through local barriers but may also increase the likelihood of LNM. Texture structure and spatial relationship features focus on describing spatial dependence and complexity between pixels. Cluster Prominence characterizes the skewness of the gray-level aggregation pattern, while Joint Entropy quantifies the complexity of spatial arrangement. In thyroid cancer with diabetes, the peritumoral tissue often exhibits increased angiogenesis, stromal remodeling, and active immune escape processes, resulting in a more complex spatial structure. On imaging, this complexity may be manifested as higher texture skewness and entropy values. These values may indicate that the boundary between the tumor and surrounding tissue is more indistinct and the tissue arrangement is more disordered, thereby reflecting stronger invasive and metastatic potential. Therefore, the results of this study may serve as an interpretable radiomics-based LNM prediction model.

Despite multicenter validation and favorable predictive performance, this study has several limitations. First, the retrospective design may have introduced selection bias, indicating the need for prospective external validation studies. Second, although data were collected from multiple centers, the sample size limited some subgroup analyses, particularly comparisons of LNM between diabetic and non-diabetic patients. Third, radiomics feature extraction was based mainly on manual ROI delineation, which may be affected by interobserver variability; automated segmentation may improve consistency. Fourth, the present study only explored relatively narrow peritumoral ranges, and wider margins were excluded mainly due to low ICC and poor reproducibility. Therefore, the generalizability and robustness of the conclusion regarding the optimal peritumoral region are still limited. Fifth, although model interpretability was assessed using SHAP, the clinical relevance of specific model outputs requires further investigation. Future studies should perform large-scale prospective multicenter validation, adopt standardized protocols, develop AI-based automated ROI segmentation to facilitate clinical translation of the model.

## 5 Conclusion

In conclusion, a dynamic CEUS-based intratumoral and peritumoral radiomics model for the diabetic population was constructed. This model exhibits good predictive performance for LNM in thyroid cancer and may serve as an effective tool for preoperative individualized prediction of LNM in this population.

## Data Availability

The dataset is subject to institutional and ethical restrictions. The clinical and imaging data contain potentially sensitive patient information and cannot be publicly shared. Access to the dataset is therefore limited to authorized researchers and requires approval from the corresponding institutional ethics committees. De-identified data may be made available upon reasonable request and with appropriate ethical clearance. Requests to access these datasets should be directed to YD, drduan_yayang@163.com.
