# Supplementary material for: Development and validation of an intra-tumoral and peri-tumoral radiomics model based on dynamic contrast-enhanced ultrasound for predicting lymph node metastasis in type 2 diabetic patients with thyroid cancer
Source: Front Endocrinol (Lausanne). 2026 Jun 23;17:1763631. doi: 10.3389/fendo.2026.1763631 (PMC13337394; doi:10.3389/fendo.2026.1763631)
Supplement: Supplementary file 1 [file DataSheet1.docx]

***Supplementary Material***

**Supplemantary figure**


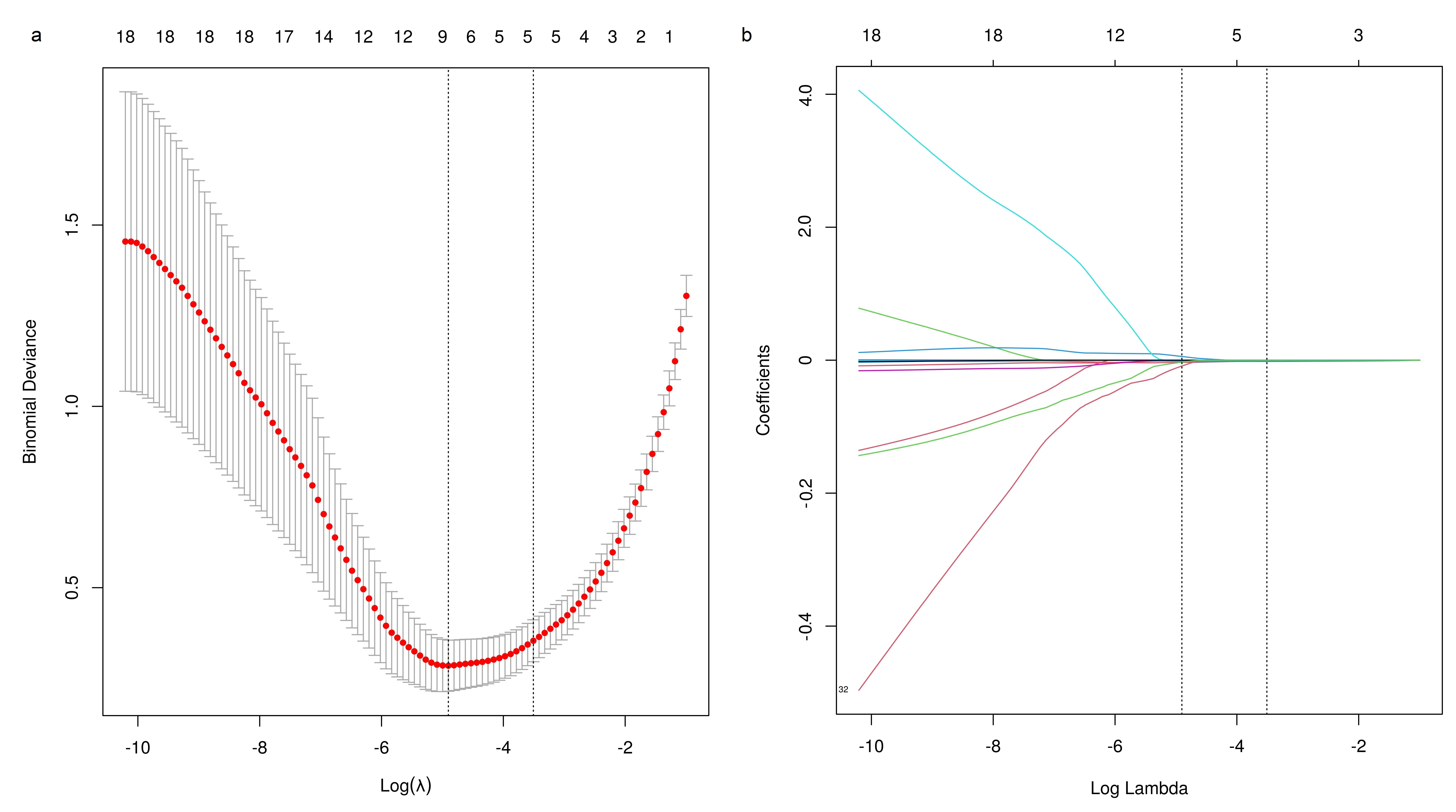


Supplemantary figure S1. Intratumoral radiomics features were filtered via the least absolute shrinkage and selection operator (LASSO) model with 10 - fold cross - validation in the training cohort. (a) Binomial deviance curves were graphed against lambda (λ). The optimal λ value was identified by the minimum criterion to achieve the lowest average binomial deviance. Bold dashed vertical lines were drawn at the optimal values based on the minimum criterion and 1 standard error of the minimum criterion, and an optimal λ value of 0.007 was chosen. (b) Coefficients of the 7 features picked out by LASSO were presented.

The intratumoral radiomics scores were calculated with the following formulas: Radiomics score = -0.024366+TTP-wavelet-HLH_glrlm_GrayLevelNonUniformity*0.818534+TTP- wavelet-

LLL_glcm_ClusterTendency*0.08276+TTP-squareroot_firstorder_Kurtosis*0.07621+TTHP

-exponential_glszm_ZoneVariance*0.03195-TTHP-wavelet-

HLL_glszm_GrayLevelNonUniformityNormalized*0.04128-TTP-wavelet-LLH_glrlm_ShortRunEmphasis*0.05427-TTP-original_gldm_DependenceNonUniformity*0.23053


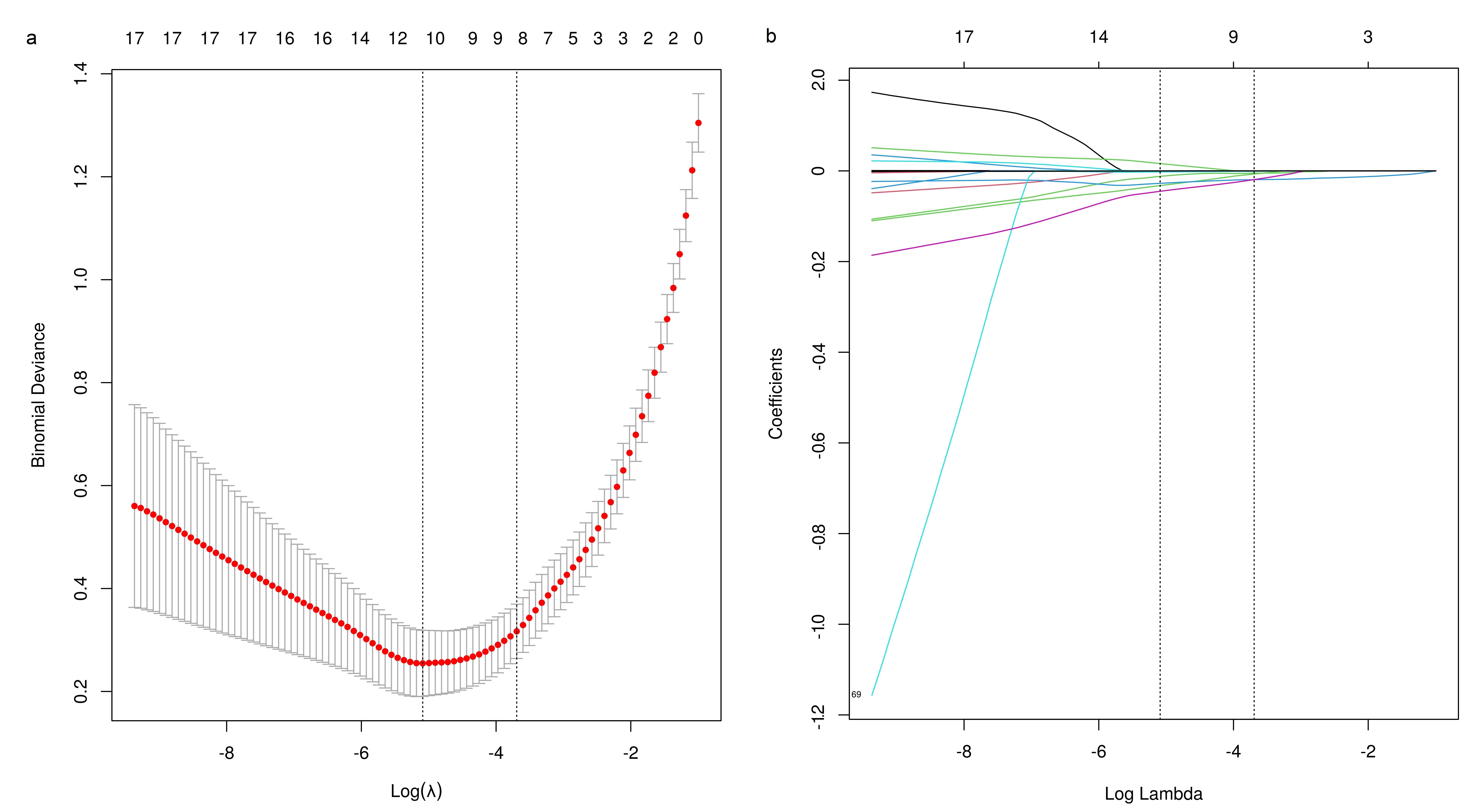
Supplemantary figure S2. Peritumoral-1mm radiomics features were filtered via the least absolute shrinkage and selection operator (LASSO) model with 10-fold cross-validation in the training cohort. (a) Binomial deviance curves were graphed against lambda (λ). The optimal λ value was identified by the minimum criterion to achieve the lowest average binomial deviance. Bold dashed vertical lines were drawn at the optimal values based on the minimum criterion and 1 standard error of the minimum criterion, and an optimal λ value of 0.006 was chosen. (b) Coefficients of the 8 features picked out by LASSO were presented.

The Peritumoral-1mm radiomics scores were calculated with the following formulas: Radiomics score=0.064932+TTP-original_glcm_SumSquares*0.020132+TTP-original_glrlm_RunPercentage*0.003127-TTP-original_glrlm_ShortRunHighGrayLevelEmphasis*0.03043-TTHP-wavelet-LLH_glrlm_LongRunLowGrayLevelEmphasis*0.03342-TTP-wavelet-HLL_glcm_Correlation*0.034758-TTP-wavelet-HLH_glszm_GrayLevelVariance*0.048124-TTHP-wavelet-HHL_gldm_DependenceNonUniformity*0.060646-TTP-wavelet-HHL_gldm_GrayLevelNonUniformity*0.070143


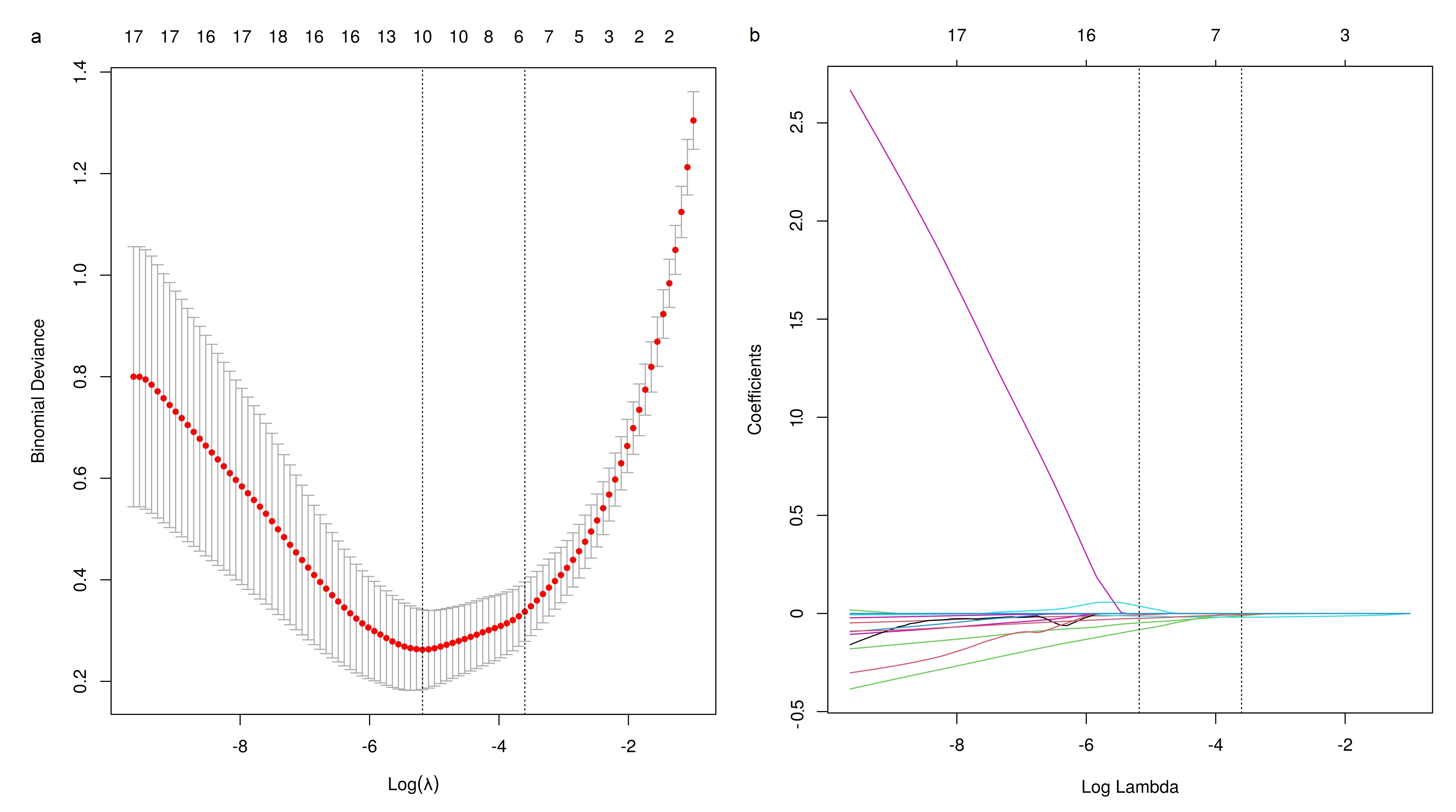


Supplemantary figure S3. Peritumoral-2mm radiomics features were filtered via the least absolute shrinkage and selection operator (LASSO) model with 10-fold cross-validation in the training cohort. (a) Binomial deviance curves were graphed against lambda (λ). The optimal λ value was identified by the minimum criterion to achieve the lowest average binomial deviance. Bold dashed vertical lines were drawn at the optimal values based on the minimum criterion and 1 standard error of the minimum criterion, and an optimal λ value of 0.006 was chosen. (b) Coefficients of the 7 features picked out by LASSO were presented.

The Peritumoral-2mm radiomics scores were calculated with the following formulas: Radiomics score=0.049263+TTHP-wavelet-LHL_gldm_GrayLevelNonUniformity*0.08471+TTHP-wavelet-HLL_glszm_GrayLevelNonUniformityNormalized*0.028247+TTHP-wavelet-HLH_glcm_ClusterProminence*0.0243-TTHP-wavelet-HHL_glcm_JointEntropy*0.038146-TTHP-wavelet-HHH_firstorder_Range*0.037263-TTHP-wavelet-LLL_firstorder_InterquartileRange*0.0592146-TTP-gradient_firstorder_10Percentile*-0.0891632

**Supplemantary text:**

**Image Feature Classification and Explanation**

**Category 1: Gray-Level Distribution and Statistical Features**

This category primarily describes the overall distribution characteristics of gray levels in an image.

- squareroot_firstorder_Kurtosis (Kurtosis: Describes the sharpness of the distribution shape)
- exponential_glszm_ZoneVariance (Zone Variance: Quantifies the dispersion of gray levels within a region)
- wavelet-HLH_glszm_GrayLevelVariance (Gray-Level Variance: Reflects the fluctuation of gray levels)
- wavelet-HHH_firstorder_Range (Range: The difference between the maximum and minimum gray levels)
- wavelet-LLL_firstorder_InterquartileRange (Interquartile Range: The range of the middle 50% of gray levels)
- gradient_firstorder_10Percentile (10th Percentile: Characteristic of low gray-level distribution)

**Category 2: Texture Uniformity and Heterogeneity**

This category mainly measures the uniformity or heterogeneity of image texture (focusing on the distribution uniformity of gray levels or regions).

- wavelet-HLH_glrlm_GrayLevelNonUniformity (Gray-Level Non-Uniformity: The inconsistency in the distribution of gray levels)
- wavelet-HLL_glszm_GrayLevelNonUniformityNormalized (Normalized Gray-Level Non-Uniformity: The normalized difference in gray-level distribution)
- original_gldm_DependenceNonUniformity (Dependence Non-Uniformity: The inconsistency in the distribution of gray-level dependence)
- wavelet-HHL_gldm_DependenceNonUniformity (Dependence Non-Uniformity in Wavelet Domain)
- wavelet-HHL_gldm_GrayLevelNonUniformity (Gray-Level Non-Uniformity in Wavelet Domain)
- wavelet-LHL_gldm_GrayLevelNonUniformity (Gray-Level Non-Uniformity in Wavelet Domain)
- wavelet-HLL_glszm_GrayLevelNonUniformityNormalized (Normalized Gray-Level Non-Uniformity in Wavelet Domain)

**Category 3: Texture Structure and Spatial Relationships**

This category mainly describes the spatial patterns of texture.

- wavelet-LLL_glcm_ClusterTendency (Cluster Tendency: The tendency of gray-level clusters to occur)
- wavelet-LLH_glrlm_ShortRunEmphasis (Short Run Emphasis: The degree of prominence of short runs)
- original_glcm_SumSquares (Sum of Squares: The weighted square of gray-level differences)
- original_glrlm_RunPercentage (Run Percentage: The proportion of runs in the image)
- original_glrlm_ShortRunHighGrayLevelEmphasis (Short Run High Gray-Level Emphasis: The prominence of high gray-level short runs)
- wavelet-LLH_glrlm_LongRunLowGrayLevelEmphasis (Long Run Low Gray-Level Emphasis: The prominence of low gray-level long runs)
- wavelet-HLL_glcm_Correlation (Correlation: The degree of linear dependence between pixels)
- wavelet-HLH_glcm_ClusterProminence (Cluster Prominence: The degree of salience of clusters)
- wavelet-HHL_glcm_JointEntropy (Joint Entropy: A measure of the randomness of pixel pairs)

Explanation of Classification Criteria

1. Category 1: Focuses on the statistical properties of gray levels themselves (such as variance, percentiles) and does not involve spatial relationships.
2. Category 2: The core is "Non-Uniformity", which directly reflects the degree of non-uniformity in the distribution of gray levels or regions in the image.
3. Category 3: Captures the spatial patterns of texture (such as run length, clustering characteristics) and relies on the spatial relationships between pixels.

This classification balances the functional similarity and calculation principles of features, ensuring that features in the same category have consistent interpretability in clinical applications (such as tumor heterogeneity assessment).

| Table S1. Performance Comparison of the Proposed Radiomics Model with Baseline Models | | | | |
| --- | --- | --- | --- | --- |
| Model | Included variables | Training cohort  AUC (95% CI) | External validation 1  AUC (95% CI) | External validation 2  AUC (95% CI) |
| Clinical baseline model | Age, Gender, Tumor size, BRAF, Multiplicity | 0.652 (0.561–0.733) | 0.628 (0.456–0.770) | 0.635 (0.472–0.771) |
| Conventional US baseline model | Shape, Margin, Echogenicity, Aspect ratio, Calcification, Vascularity, Two-dimensional lymph node, Two-dimensional capsular | 0.671 (0.582–0.748) | 0.643 (0.475–0.782) | 0.650 (0.491–0.783) |
| SVM | Age, Gender, Tumor size, BRAF, Multiplicity, Shape, Margin, Echogenicity, Aspect ratio, Calcification, Vascularity, Two-dimensional lymph node, Two-dimensional capsular | 0.705 (95% CI: 0.621–0.778) | 0.694 (95% CI: 0.529–0.816) | 0.692 (95% CI: 0.527–0.814) |
| RF | Age, Gender, Tumor size, BRAF, Multiplicity, Shape, Margin, Echogenicity, Aspect ratio, Calcification, Vascularity, Two-dimensional lymph node, Two-dimensional capsular | 0.712 (95% CI: 0.628–0.784) | 0.708 (95% CI: 0.544–0.827) | 0.672 (95% CI: 0.540–0.820) |
| Xgboost | Age, Gender, Tumor size, BRAF, Multiplicity, Shape, Margin, Echogenicity, Aspect ratio, Calcification, Vascularity, Two-dimensional lymph node, Two-dimensional capsular | 0.726 (95% CI: 0.645–0.795) | 0.715 (95% CI: 0.553–0.833) | 0.703 (95% CI: 0.564–0.838) |
| Our combined radiomics model | Intra-tumoral + 2 mm peri-tumoral CEUS radiomics scores | 0.930 (0.876–0.964) | 0.907 (0.796–0.968) | 0.865 (0.739–0.941) |
| CI: Confidence Interval | | | | |
